# Characterization of gut microbiota dysbiosis in breast cancer patients

**DOI:** 10.1007/s12282-025-01782-8

**Published:** 2025-10-02

**Authors:** Mai Yamada, Makoto Kubo, Kazuhisa Kaneshiro, Masaya Kai, Takafumi Morisaki, Saori Hayashi, Yurina Ochiai, Yo Sato, Kimihisa Mizoguchi, Yuka Takao, Akiko Arimura, Masafumi Nakamura

**Affiliations:** 1 Department of Surgery and Oncology Graduate School of Medical Sciences, Kyushu University, 3-1-1 Maidashi Higashi-Ku, Fukuoka, 812-8582 Japan; 2 Department of Breast Surgical Oncology, Kyushu University Hospital, Fukuoka, Japan

**Keywords:** Gut microbiome, Breast cancer, Dysbiosis, RNA sequencing, Lifestyle factor

## Abstract

**Background:**

While lifestyle factors are known to be associated with breast cancer development, the potential role of the gut microbiome, which is influenced by lifestyle, as a risk factor is not well understood. We conducted a comparative analysis of the intestinal microbiota between healthy individuals and breast cancer patients to investigate the potential impact of gut microbiome composition on breast cancer development. This study aimed to explore the role of intestinal microbial communities in breast cancer pathogenesis.

**Methods:**

We conducted a comparative analysis of fecal 16S rRNA amplicon sequencing data from 100 individuals in the general population and 79 breast cancer patients. We investigated the differences between the two groups in terms of relative abundance, absolute quantity, diversity, and functionality of the gut microbiota.

**Results:**

Breast cancer groups showed higher levels of Firmicutes and lower levels of Bacteroidota at the phylum level, and an increase in Fusobacteriota was found in the human epidermal growth factor receptor 2 (HER2)-negative breast cancer group. Additionally, certain genera were more or less common in breast cancer groups at the genus level. The study also indicated lower gut microbiota diversity and loss of heterogeneity in breast cancer groups and reduced functional genes and pathways.

**Conclusion:**

Compared to the general population, breast cancer patients exhibited a distinct dysbiosis in their gut microbiota. Further investigation is warranted to determine if this dysbiotic state, linked to a predicted downregulation of functional pathways critical for homeostasis, plays a role in breast cancer development.

## Introduction

Breast cancer affected over 2.3 million people in 2020, with projections indicating more than 3 million cases and 1 million deaths annually by 2040, partly due to lifestyle factors like obesity and lack of exercise [1].

Recent findings highlight the impact of gut and oral microbiotas, linked to lifestyle habits, on systemic diseases and cancer, known as the term of oncobiome [2].

Reduced gut microbiota diversity can impair immune function and systemic homeostasis, influencing cancer progression and treatment efficacy [3, 4]. Some studies have explored the relationship between breast cancer and gut microbiota [5–8]. It has long been known that gut microbiota deconjugate estrogens, affecting estrogen levels [9]. However, recent studies have established a link between dietary habits, such as high-fat, high-fiber, and Mediterranean diets, and breast cancer, showing that these diets directly influence gut microbiota [10, 11]. Furthermore, mounting evidence indicates that Fusobacterium, a periodontal bacterium, is associated with gastrointestinal cancer [12], and the lifestyle-related intestinal environment may contribute to the increasing number of breast cancer cases.

While some studies have examined the link between gut microbiota and breast cancer risk [6], consensus is lacking, and applicability to humans remains uncertain due to variations in microbiota by geography, race, and living environment.

This study compares the pretreatment fecal flora of breast cancer patients with the general female population to assess whether specific intestinal microbiota influence breast cancer development or progression, revealing significant differences in bacterial species, functional genes and pathways. Our data suggest that these differences may be associated with the biological behavior of breast cancer.

## Patients and methods

### Study subjects and data collection

We analyzed fecal samples and metadata from two Japanese cohorts. The public control (PC) cohort consisted of community volunteers participating in a health and nutrition study conducted by the National Institutes of Biomedical Innovation, Health and Nutrition (NIBIOHN). From NIBIOHN’s public data, we randomly selected 100 women aged 31–79 years, which corresponds to the age range most susceptible to breast cancer. Fecal samples were collected from five different cities in Japan, with 20–30 samples randomly selected from each of Tokyo, Niigata, Osaka, and Yamaguchi (Supplementary Table 1) [13, 14]. For comparison, the breast cancer (BC) cohort, recruited specifically for this study, included 79 breast cancer patients who received treatment at Kyushu University Hospital between April 2021 and March 2023. Patients undergoing long-term antibiotic treatment, using immunosuppressants, or who were pregnant were excluded from the study. This research adhered to the principles of the Declaration of Helsinki and was approved by the Ethics Committee of Kyushu University Hospital (Approval No. 23030-00). Participants were allowed to withdraw from the study at any time. In the BC cohort, seven patients had ductal carcinoma in situ and two had microinvasive breast cancer (< 1 mm invasion), leading to categorization into Ductal Carcinoma in Situ (DCIS) dominant (≦ T1mic) and others into invasive breast cancer (≧ T1a) (IBC) groups. Patient data were sourced from pathology results and medical records. One of the 70 invasive breast cancer cases involved bilateral cancer with different subtypes and was excluded from subtype analysis. Among the remaining 69 cases, patients were classified as luminal type (hormone recepter [HR] positive, human epidermal growth factor receptor 2 [HER2] negative), luminal-HER2 type (HR positive, HER2 overexpressed), pure HER2 type (HR negative, HER2 overexpressed), and triple negative type (HR and HER2 negative) (Table 1).

**Table 1:** Clinical characteristics of the breast cancer and public control cohorts

	Breast Cancer	Public Control
	*N* = 79	*N* = 100
Age at diagnosis, years, mean (range)	52.8 (31–76)	57.9 (35–79)
*T-stage *(*excluding Bil. BC*)		
*DCIS dominant*		
Tis	7	–
T1mic	2	–
IBC		
T1	40	–
T2	24	–
T3	5	–
T4	1	–
*Lymph node status*		
Positive	20	–
Negative	59	–
*ER status*		
Positive	51	–
Negative	18	–
*Subtype (IDC = 69 excluding Bil. BC)*		
Luminal type	39	–
Luminal HER2 type	12	–
HER2 type	6	–
Triple negative type	12	–
*HER2 status (IDC = 69 excluding Bil. BC)*		
Positive	18	–
Negative	51	–
*Menopausal status*		
Pre-menopause	25	–
Post-menopause	54	–
Unknown	–	100
*BMI*		
BMI ≤ 22	37	–
22 < BMI ≤ 25	18	–
25 < BMI ≤ 30	18	–
BMI > 30	6	–
Unknown	–	100

Bil. *BC* bilateral breast cancer, *DCIS* ductal carcinoma in situ, early breast cancer, *IBC* invasive breast cancer, *ER* estrogen receptor, *IDC* invasive ductal carcinoma, *HER2* human epidermal growth factor receptor 2, *BMI* body mass index

### Fecal collection and 16S rRNA amplicon sequencing

Stool samples were self-collected from patients using a stool collection tube from TechnoSuruga Labs, Inc (Shizuoka, Japan) before the initiation of any treatment.The DNA was extracted by an automated DNA isolation system (GENE PREP STAR PI-480, KURABO, Osaka, Japan). V3–V4 regions of bacterial and archaeal 16S rRNA were amplified using Pro341F/Pro805R primers and the dual-index method [15, 16]. Barcoded amplicons were paired-end sequenced on a 2 × 301-bp cycle using the MiSeq system with MiSeq Reagent Kit version 3 (600 Cycle) chemistry.

### Metagenomic analysis of gut microbiota

Amplicon sequence reads were processed using the QIIME2 pipeline (ver. 2021.2) [17]. Sequence denoising, paired-end read merging, and chimera filtering were conducted with the DADA2 (divisive amplicon denoising algorithm 2) [18] method, using the following parameters: -p-trim-left-f 30, -p-trim-left-r 30, -p-trunc-len-f 260, -p-trunc-len-r 220, and truncating reads where the median quality score was below 30. Operational taxonomic units (OTUs) were clustered at a 97% similarity threshold. Taxonomy was assigned using the SILVA 138–99-classifier [19], focusing on 16S rRNA OTUs from the V3–V4 region. Differential abundance analysis was performed with ANCOM to identify significant differences in bacterial flora at phylum and genus levels [20]. PICRUSt2 (phylogenetic investigation of communities by reconstruction of unobserved states) pipeline (https://github.com/picrust/picrust2) [21], was used for functional prediction, including KEGG Orthology (KO) pathway analysis, based on 16S rRNA sequencing data [22].

### Statistical analysis

Statistical analyses were conducted using Prism 7 (GraphPad Software Inc.). To adjust for multiple comparisons, Benjamini and Hochberg’s FDR correction was applied, with significance set at FDR *p*-value ≤ 0.05. Phylum and genus abundances were evaluated using the Kruskal–Wallis test, while alpha-diversity differences were assessed with ANOVA. PERMANOVA, a non-parametric ANOVA, tested beta-diversity differences. Alpha-diversity was measured using chao1, observed features, Faith’s phylogenetic diversity, Shannon, Simpson, and Pielou’s evenness indexes. Beta-diversity was assessed using Bray–Curtis, Jaccard, and weighted unifraction distances, visualized via PCoA. Community composition differences were evaluated using an analysis of similarity with Bonferroni’s correction for multiple comparisons. Paired analyses were conducted where applicable.

## Results

### Differences in relative abundances of gut microbiota between the public cohort and breast cancer patients

**Fig. 1:**
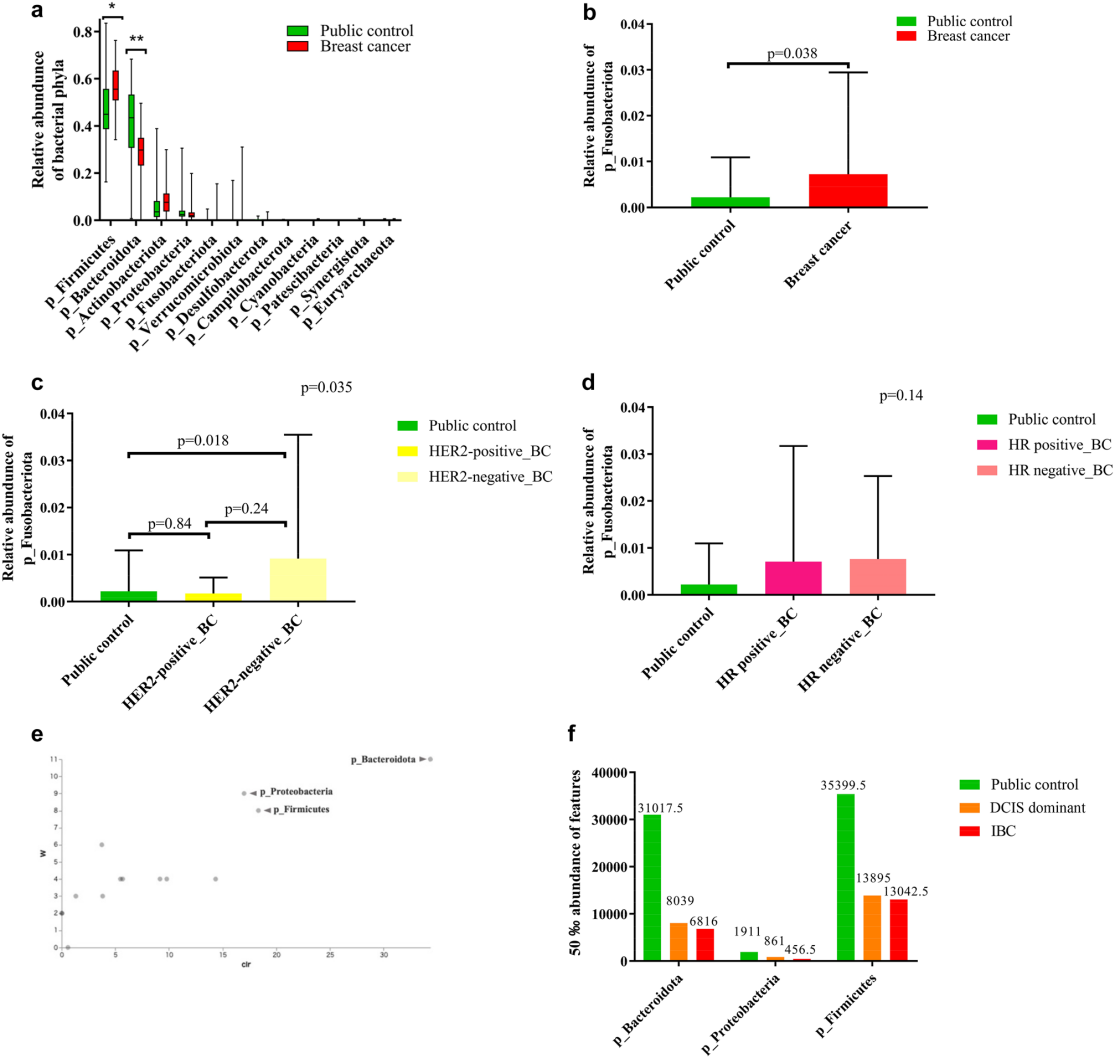

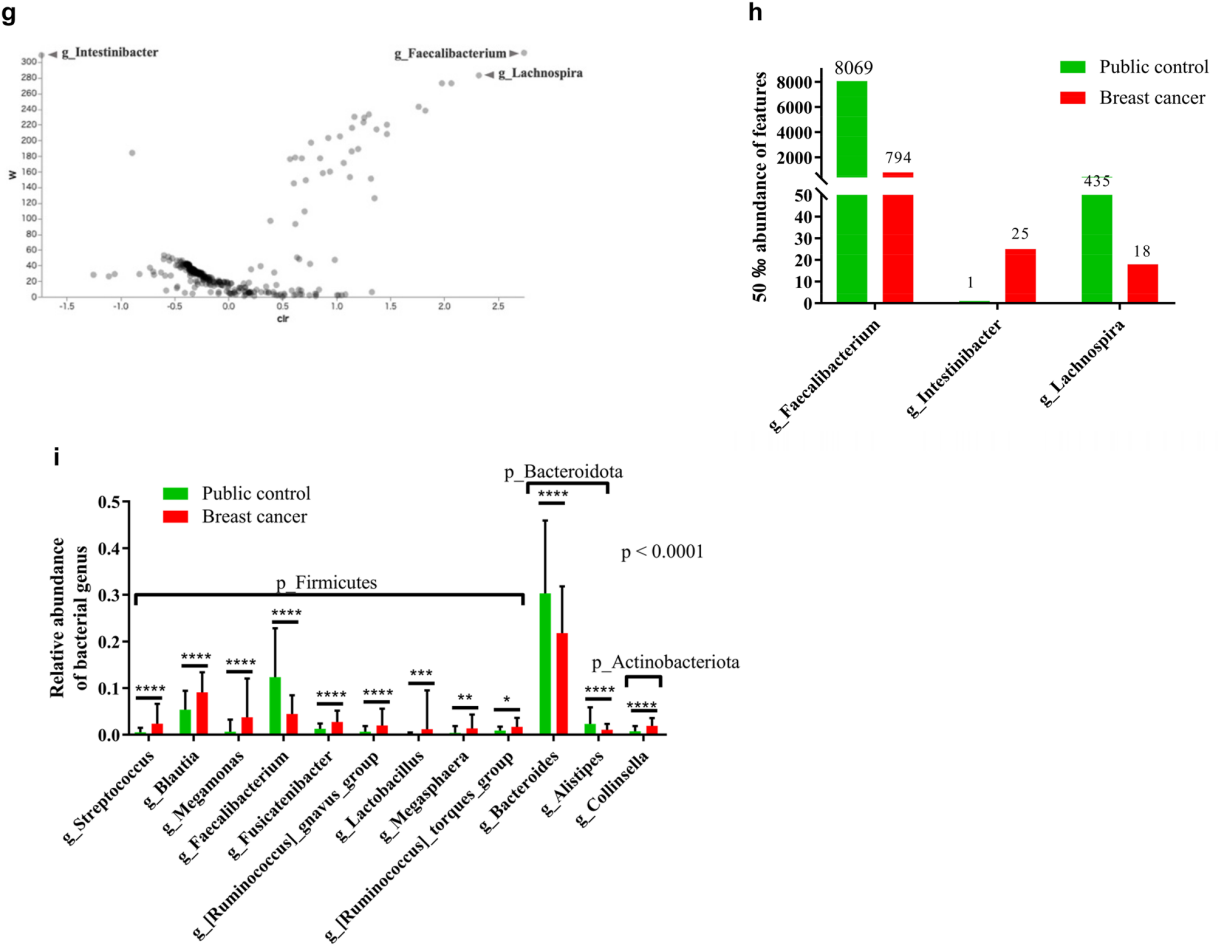
Gut microbiota at the phylum and genus level. **a** Box plots of the relative abundances of bacterial phyla in the public cohort and breast cancer cohort. *, **p < 0.0001 (multiple t-test and Bonferroni–Dunn test). **b**, **c**, **d** Bar graph of the relative abundance of the Fusobacteroidota phylum. **e**, **f** Significantly differentially abundant microbial phyla identified by ANCOM between PC, DCIS dominant, and IBC groups and the 50th percentile values. **g**, **h** Significantly differentially abundant microbial genera identified by ANCOM between public cohort and breast cancer groups and the 50th percentile values. Clr (x-axis) is the measure of the effect size difference for a particular microbiome classification among the groups. W-statistic (y-axis) is the strength of the ANCOM test for the number of species included in the classification tested. **i** Relative abundance of bacterial genera in public cohort and breast cancer cohorts. Data are shown as the mean with SD. *p* < 0.0001 (*p*-value summary of MANOVA and the Wilks λ test). *p = 0.025, **p = 0.0019, ***p < 0.0001, ****p < 0.00001 (multiple *t*-test and Bonferroni–Dunn test)

The relative abundances of the fecal microbiome at the phylum level were compared between the BC cohort (DCIS dominant and IBC) and the PC cohort. Both cohorts were dominated by Firmicutes and Bacteroidota. The relative abundance of Firmicutes was significantly higher in the BC cohort than in the PC cohort (*p* < 0.0001), while Bacteroidota was significantly lower in the BC cohort (*p* < 0.0001) (Fig. 1a).

The relative abundance of Fusobacteriota at the phylum level was significantly higher in the BC cohort compared to the PC cohort (*p* = 0.038) (Fig. 1b). No significant difference in Fusobacteriota abundance was observed between the PC cohort and HER2-positive BC group (*p* = 0.84). Significant differences were found between the PC cohort and the HER2-negative BC group (*p* = 0.018), as well as the HR-positive/HER2-negative (LUM) group (*p* = 0.038) (Fig. 1c; S1a). Comparisons between the PC cohort and HR-positive or HR-negative BC groups showed no significant differences (Fig. 1d).

ANCOM analysis [20] revealed significant differences in the microbiome composition at the phylum level among the PC, DCIS dominant, and IBC groups, specifically in Bacteroidota, Proteobacteria, and Firmicutes (Fig. 1e). The 50th percentile abundance of features was significantly decreased as breast cancer progressed, compared with the PC cohort (Fig. 1f). As the genus level, ANCOM analysis identified a significant decrease in the abundance of butyrate-generating bacteria Feacalibacterium and Lachnospira in the BC cohort compared to the PC cohort, whereas Intestinibacter abundance was increased (Fig. 1g, h). Significant differences in the abundance of Feacalibacterium and Intestinibacter were observed among the DCIS dominant, IBC, and PC groups (Fig. S1b, S1c).

At the genus level, nine genera of Firmicutes, two genera of Bacteroidota, and one genus of Actinobacteria showed significant differences between the BC and PC cohorts. Within the Firmicutes phylum, Feacalibacterium [23, 24] was significantly more abundant in the PC cohort, while the other genera were more abundant in the BC cohort. Within the Bacteroidota phylum, Bacteroides and Alistipes [25] were significantly more abundant in the PC cohort. In the Actinobacteriota phylum, Collinsella [26] was significantly more abundant in the BC cohort (Fig. 1i).

### Difference in microbiota diversity between the public cohort and breast cancer patients

**Fig. 2:**
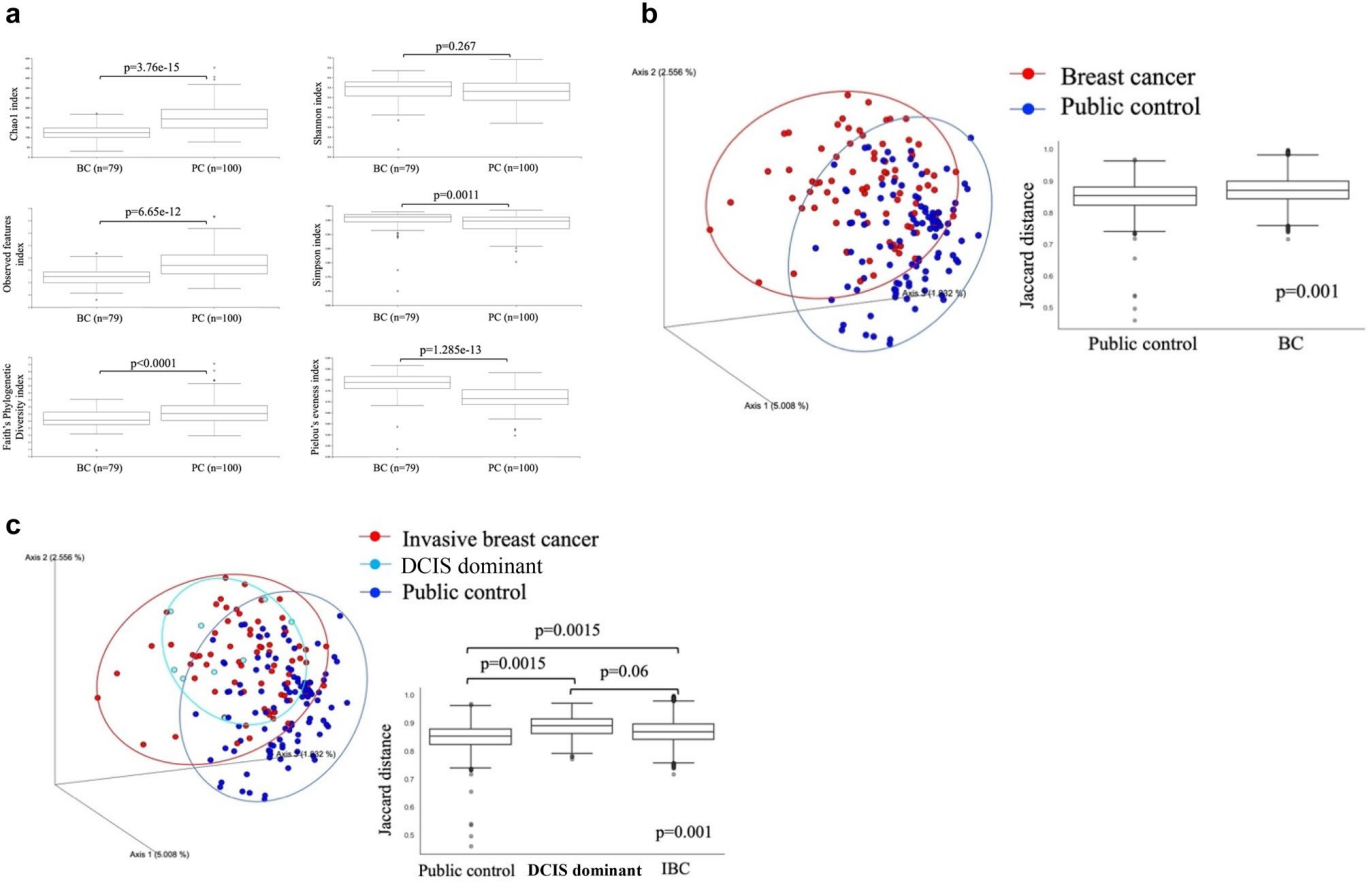
Microbiota diversity analysis among public control and breast cancer patients. **a** Box plots of the six indicators of alpha-diversity between breast cancer (BC) and public control (PC) cohorts. P-value (pairwise Kruskal–Wallis test). **b**, **c** Beta-diversity analysis. Principal coordinate analysis plots and box plot of Jaccard distance analysis of the microbiota in each group. *p* = 0.001 (represents the *p*-value summary of PERMANOVA and each correlation by pairwise PERMANOVA)

Alpha-diversity between BC and PC cohorts was evaluated using three indicators reflecting species richness (chao1, observed features, and Faith’s phylogenetic diversity indexes). The BC cohort exhibited significantly lower values in all three richness-related indicators. In contrast, indicators reflecting the evenness of bacterial species distribution (Shannon, Simpson, and Pielou’s evenness indexes) showed significantly higher values in the BC cohort compared to the PC cohort, indicating a more uniform distribution of a smaller number of bacterial species in the BC cohort (Fig. 2a).

A comparable pattern was observed in the comparison among the PC, DCIS dominant and IBC groups. The DCIS dominant group showed lower bacterial richness than the IBC group, while the IBC group exhibited higher evenness compared to the DCIS dominant group (Fig. S2a).

Beta-diversity, which quantifies differences in microbial composition among groups, was also assessed. Significant differences in beta-diversity were observed between the BC and PC cohorts (Fig. 2b; Fig. S2b, S2c). The degree of dissimilarity in beta-diversity between the PC and DCIS dominant groups was greater than that between the PC and IBC groups (Fig. 2c; Fig. S2d, S2e).

### Associations of gene expression, enzyme levels, and working pathways with microbiome dysbiosis

**Fig. 3:**
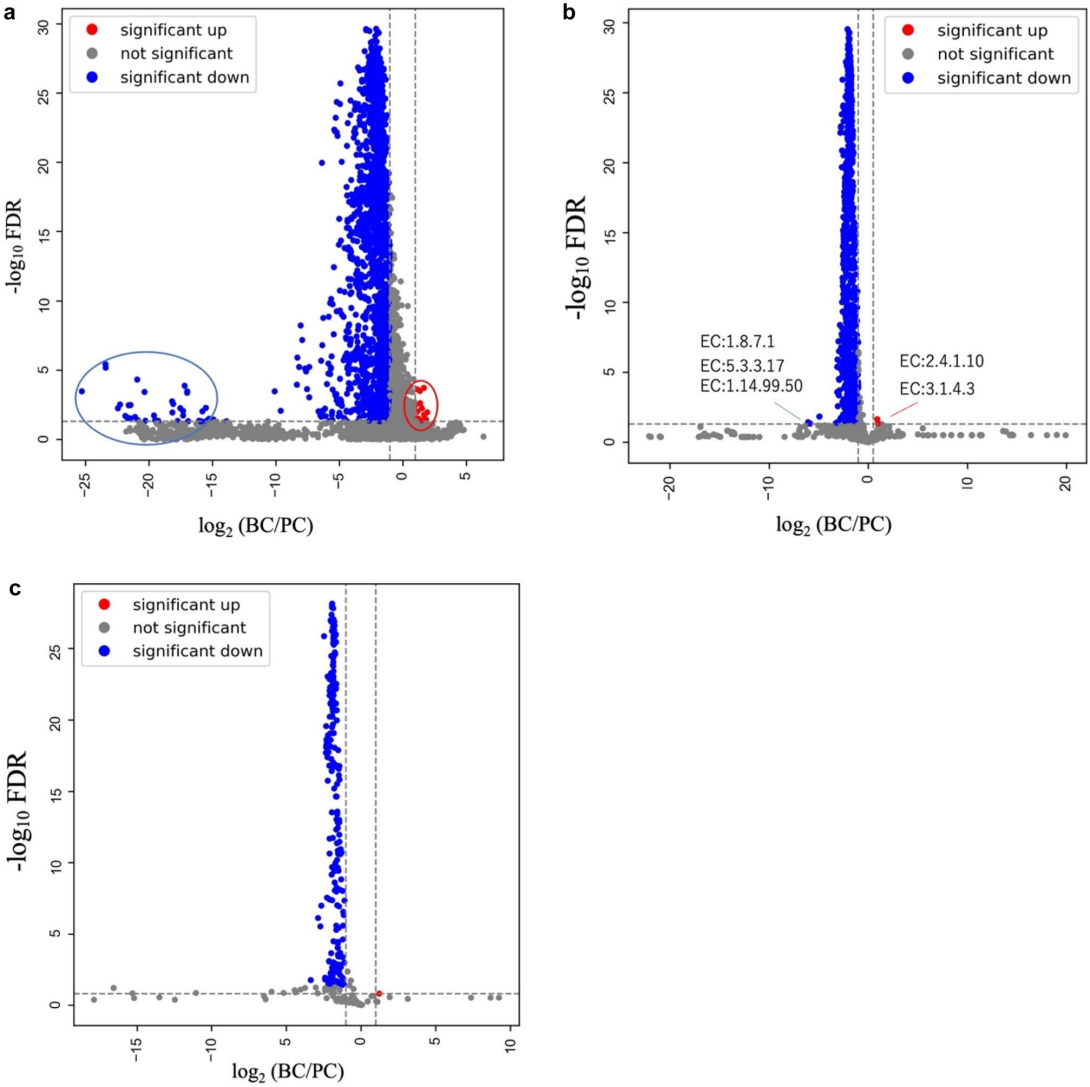
Predictive analysis of functional differences on the basis of microbiota metagenomic analysis. **a** Kyoto Encyclopedia of Genes and Genomes Orthology metagenome analysis of PC vs. BC cohorts. Volcano plot of the differential expression of genes vs. false discovery rate (FDR) is shown. The threshold for significant differences was set at – 1 > log (fold change; BC/PC) > 1 and FDR < 0.05. **b** EC metagenome analysis of PC vs. BC cohorts. Volcano plot of the relationship between differential expression of reference enzymes corresponding to ortholog genes and FDR. The threshold for significant differences was set at – 1 > log (fold change; BC/PC) > 0.5 and FDR < 0.05. **c** Pathway abundance analysis of PC vs BC cohorts. Volcano plot of the relationship between differential expression of reference pathways corresponding to ortholog genes and FDR. The threshold for significant differences was set at – 1 > log (fold change; BC/PC) > 1 and FDR < 0.05

Metagenomic analysis of gut microbiota was followed by functional prediction using PICRUSt2 [21], based on the obtained sequence information. Mapped to the Kyoto Encyclopedia of Genes and Genomes (KEGG) database [22], changes in metabolic pathways were examined. The PICRUSt2 analysis identified 6539 orthologous genes, of which 3272 (50.0%) showed statistically significant differences in predicted expression levels between BC and PC cohorts. (FDR < 0.05). These genes were associated with 292 pathways, including the general metabolic pathway (1088 orthologous genes) and biosynthesis of secondary metabolites (401 orthologous genes). Among these genes, 3107 (95%) exhibited a more than two-fold decrease in predicted expression in the BC group compared with the PC group, while 165 genes (5%) showed an increase of more than twofold (Fig. 3a and Table S2).

Among 1,932 identified microbial enzymes, 1236 (64%) showed statistically significant differences in predicted abundance between the BC and PC groups (FDR < 0.05). The majority of these enzymes exhibited reduced expression in the BC group, with no enzymes showing a more than two-fold increase. However, levansucrase (EC 2.4.1.10), involved in sucrose metabolism, and phospholipase C (EC 3.1.4.3), which hydrolyzes phospholipids in cell membranes, exhibited increases of approximately 1.8-fold. Of the 1,208 enzymes with significant decreases to less than one-eighth of the PC group levels, 14 showed particularly large reductions. These included sulfite reductase (ferredoxin) (EC 1.8.7.1), involved in selenoamino acid and sulfur metabolisms; trans-2,3-dihydro-3-hydroxyanthranilate isomerase (EC 5.3.3.17); and gamma-glutamyl hercynylcysteine S-oxide synthase (EC 1.14.99.50), which participates in the biosynthesis of ergothioneine, a compound with antioxidant properties (Fig. 3b and Table S3).

Among the 369 KEGG pathways analyzed, 283 pathways (76.7%) showed significant differences in predicted abundance between the BC and PC groups (FDR < 0.05). Of these, 280 pathways (75.9%) exhibited significantly lower abundance in the BC group, with no pathway showing a more than two-fold increase. In 55 pathways (19.4%), predicted abundance decreased to less than one-fourth of PC group levels. These included pyrimidine deoxyribonucleosides salvage (PWY-7199), CMP-legionaminate biosynthesis I (PWY-6749), sucrose degradation IV via sucrose phosphorylase (PWY-5384), the superpathway of sulfate assimilation and cysteine biosynthesis (SULFATE-CYS-PWY), fatty acid elongation – saturated (FASYN-ELONG-PWY), nitrate reduction VI (assimilatory) (PWY490-3), and the incomplete reductive TCA cycle (P42-PWY), all showing abundance levels reduced to 1/2000 or less compared to the PC group. Among the 55 most strongly decreased pathways, several were related to shared biological processes, including biosynthesis pathways of ubiquinol (PWY-5855, PWY-5856, PWY-5857, PWY-6708, UBISYN-PWY), pathways involved in fatty acid metabolism (FASYN-ELONG-PWY, FASYN-INITIAL-PWY, FAO-PWY, PWY-7094), L-arginine metabolism (PWY-5154, ARGDEG-PWY, ARNARGDEG-PWY, AST-PWY), and pathways associated with the TCA cycle (P42-PWY, TCA-GLYOX-BYPASS) (Fig. 3c and Table S4).

### Effect of microbiome differences on breast cancer subtypes

**Fig. 4:**
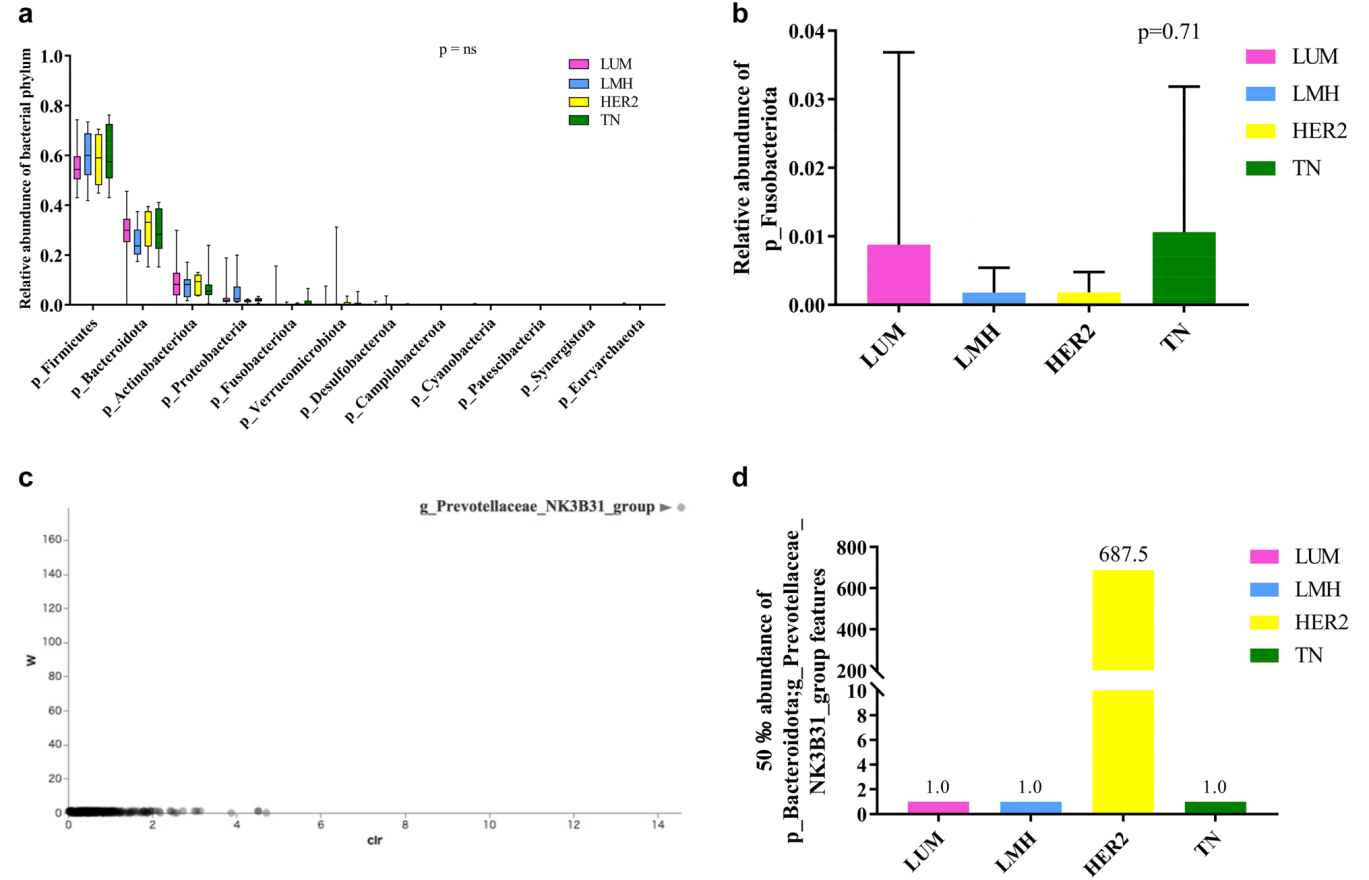
Gut microbiome metagenome analysis of breast cancer subtypes. **a** Relative frequency of each BC subtype at the phylum level. ns = not significant (MANOVA and the Wilks λ test). Bar scale shows minimum and maximum values. **b** Relative abundance of the Fusobacteroidota phylum in each breast cancer subtype. *ns* not significant (the *p*-value summary of one-way ANOVA). **c**, **d** Significantly differentially abundant microbial taxa identified by ANCOM among each BC subtype and the 50th percentile values. Clr (x-axis) is the measure of the effect size difference for a particular genus among the four subtypes. W-statistic (y-axis) is the strength of the ANCOM test for the number of species included in the genera tested

In the BC cohort, the overall composition of intestinal bacteria differed significantly from that in the PC cohort. To assess whether these differences were also observed among breast cancer subtypes, bacterial compositions were compared across subtypes. At the phylum level, no statistically significant differences were observed among the subtypes (Fig. 4a). Although the relative abundance of the Fusobacterium phylum appeared lower in the HER2-negative group, the difference was not statistically significant (Fig. 4b). Quantitative comparisons at the genus level using ANCOM analysis revealed that the genus *Prevotellaceae_NK3B31_group*, belonging to the Bacteroidota phylum, was significantly more abundant in the pure HER2 subtype group (Fig. 4c, d). No statistically significant differences in microbial diversity were observed among subtypes when analyzed in relation to various clinicopathological factors. However, alpha-diversity assessed by richness-based indicators (Chao1 and observed features) tended to be higher in estrogen receptor (ER)-positive and progesterone receptor (PgR)-positive groups compared to negative groups (Table 2). When stratified by BMI, the Peilou_Evenness index showed a trend toward higher alpha-diversity in individuals with BMI < 25 (Table 2). Beta-diversity analysis revealed statistically significant differences between premenopousal and postmenopausal women (*p* = 0.03), as well as between patients with and without lymph node metastasis (*p* = 0.009) (Table 3). No significant differences were detected in predicted gene (KO) or enzyme (EC) abundance, or in pathway-level metagenomic predictions (data not shown).

**Table 2:** Alpha diversity by clinicopathologic factors in the breast cancer cohort

Alpha diversity	Chao1	faith_pd	observed_feature	Shannon	Pielou_evenness	Simpson
Subtype (LUM, LMH, HER2, TN)	0.3	0.59	0.33	0.58	0.46	0.56
Stage (1, 2, 3)	0.54	0.31	0.62	0.74	0.61	0.74
HER2	0.76	0.58	0.74	0.76	0.26	0.42
T stage (1–4)	0.73	0.45	0.77	0.73	0.68	0.56
N stage (N0–N3)	0.71	0.45	0.74	0.61	0.15	0.48
LN met	0.198	0.13	0.24	0.59	0.77	0.9
ER	**0.056**	0.19	**0.065**	0.2	0.7	0.34
PgR	**0.083**	0.2	0.1	0.49	0.64	0.88
BMI	0.45	0.44	0.49	0.63	**0.097**	0.17
cutoff 25						
BMI	0.49	0.35	0.59	0.91	0.16	0.34
cutoff 18, 22, 25						
Menstruation	0.58	0.7	0.62	0.45	0.17	0.75
Alcohol (yes/no/occasional)	0.49	0.34	0.46	0.95	0.9	0.9
Smoking	0.43	0.21	0.4	0.32	0.3	0.58

*LUM* luminal type as estrogen receptor-positive and human epidermal growth factor receptor 2-negative breast cancer, *LMH* luminal-HER2 type as hormone receptor-positive breast cancer with human epidermal growth factor receptor 2 overexpressionz, *HER2* pure HER2 type as hormone receptor-negative breast cancer with human epidermal growth factor receptor 2 overexpression, *TN* triple negative breast cancer, *LN met* lymph node metastasis, *ER* estrogen receptor, *PgR* progesterone receptor, *BMI* body mass index

Bold values indicates values that showed a trend toward significance (0.05 ≤ p 0.1) are indicated in bold

**Table 3:** Beta diversity by clinicopathologic factors in the breast cancer cohort

Beta diversity	Bray Curtis (p or q value)	Jaccard (p or q value)	Weighted–unifrac (p or q value)
Subtype	LUM vs LMH 0.892 Total 0.834	LUM vs LMH 0.812 Total 0.72	LUM vs LMH 0.708 Total 0.662
	LUM vs TN 0.908	LUM vs TN 0.812	LUM vs TN 0.763
	LUM vs HER 0.892	LUM vs HER 0.812	LUM vs HER 0.763
	HER vs LMH 0.892	HER vs LMH 0.812	HER vs LMH 0.708
	HER vs TN 0.892	HER vs TN 0.812	HER vs TN 0.708
	LMH vs TN 0.892	LMH vs TN 0.812	LMH vs TN 0.708
Stage (1–3)	Stage 1 vs 2: 0.425 Total 0.087	Stage 1 vs 2: 0.309 Total 0.087	Stage 1 vs 2: 0.338 Total 0.04
	Stage 1 vs 3: 0.158	Stage 1 vs 3: 0.119	Stage 1 vs 3: **0.063**
	Stage 2 vs 3: 0.111	Stage 2 vs 3: 0.119	Stage 2 vs 3: **0.093**
HER2	0.563	0.712	0.647
T stage (1–4)	Stage 1 vs 2: 0.654 Total 0.381	Stage 1 vs 2: 0.702 Total 0.402	Stage 1 vs 2: 0.484 Total 0.26
	Stage 1 vs 3: 0.654	Stage 1 vs 3: 0.702	Stage 1 vs 3: 0.484
	Stage 1 vs 4: 0.654	Stage 1 vs 4: 0.702	Stage 1 vs 4: 0.392
	Stage 2 vs 3: 0.654	Stage 2 vs 3: 0.702	Stage 2 vs 3: 0.484
	Stage 2 vs 4: 0.804	Stage 2 vs 4: 0.816	Stage 2 vs 4: 0.860
	Stage 3 vs 4: 0.823	Stage 3 vs 4: 0.816	Stage 3 vs 4: 1.000
N stage (N0–N3)	N0 vs N1: 1.0	N0 vs N1: 1.0	N0 vs N1: 0.120
	N0 vs N1mic: 1.0	N0 vs N1: mic 1.0	N0 vs N1mic: 0.604
	N0 vs N2: 1.0	N0 vs N2: 1.0	N0 vs N2: 0.873
	N0 vs N3: 1.0	N0 vs N3: 1.0	N0 vs N3: 0.604
	N1 vs N1mic: 1.0 Total 0.857	N1 vs N1mic: 1.0. Total 0.656	N1 vs N1mic: 0.873. Total 0.165
	N1 vs N2: 1.0	N1 vs N2: 1.0	N1 vs N2: 0.302
	N1 vs N3: 1.0	N1 vs N3: 1.0	N1 vs N3: 0.873
	N1mic vs N2: 1.0	N1mic vs N2: 1.0	N1mic vs N2: 0.822
	N1mic vs N3: 1.0	N1mic vs N3: 1.0	N1mic vs N3: 0.650
	N2 vs N3: 1.0	N2 vs N3: 1.0	N2 vs N3: 0.286
ER	0.812	0.371	0.793
PgR	0.627	0.569	0.79
BMI cutoff 25	0.252	0.142	0.911
BMI	< 18 vs 18–25: 0.69	< 18 vs 18–25: 0.527	< 18 vs 18–25: 0.809
cutoff 18, 22, 25	< 18 vs 25–30: 0.69	< 18 vs 25–30: 0.527. Total 0.257	< 18 vs 25–30: 0.877 Total 0.625
	< 18 vs > 30: 0.69	< 18 vs > 30: 0.617	< 18 vs > 30: 0.809
	18–25 vs 25–30: 0.69	18– 25 vs 25–30: 0.527	18–25 vs 25–30: 0.527
	18–25 vs > 30: 0.69	18– 25 vs > 30: 0.527	18–25 vs > 30: 0.527
	25–30 vs > 30: 0.69	25– 30 vs > 30: 0.699	25– 30 vs > 30: 0.699
Menstruation	**0.03**	0.142	0.261
LN-met	0.418	0.236	**0.009**
Alcohol	No vs Occasional: 0.697	No vs Occasional: 0.525	No vs Occasional: 0.826
	No vs Yes 0.680: Total 0.982	No vs Yes: 0.525 Total 0.413	No vs Yes: 0.826 Total 0.855
	Occasional vs Yes: 0.680	Occasional vs Yes: 0.525	Occasional vs Yes: 0.826
Smoking	**0.051**	0.108	0.495

*LUM* luminal type as estrogen receptor-positive and human epidermal growth factor receptor 2-negative breast cancer, *LMH* luminal-HER2 type as hormone receptor-positive breast cancer with human epidermal growth factor receptor 2 overexpressionz, *HER2* pure HER2 type as hormone receptor-negative breast cancer with human epidermal growth factor receptor 2 overexpression, *TN* triple negative breast cancer, *LN met* lymph node metastasis, *ER* estrogen receptor, *PgR* progesterone receptor, *BMI* body mass index

## Discussion

The relationship between gut microbiota and breast cancer has been increasingly highlighted, with multiple studies supporting the involvement of gut microbes in breast cancer development and progression [7]. However, the gut microbiota is a highly dynamic entity that varies depending on environment, diet, and ethnicity, raising concerns about the universal applicability of existing findings [27]. In this study, we aimed to predict how the composition and functional capacity of gut microbiota in Japanese women may influence the onset of breast cancer by comparing the metagenomic profiles of healthy individuals and breast cancer patients.

Our results identified several differences in the gut microbiota profiles between the two groups. Notably, the abundance of *Faecalibacterium* and *Lachnospira*, both known to produce butyrate and contribute to anti-inflammatory and anti-tumorigenic effects, was significantly lower in the breast cancer group [23, 24]. Conversely, the relative abundance of *Blautia*, which has been reported to increase in advanced breast cancer cases and may be involved in inflammatory regulation and disease progression, was higher in the breast cancer group [31, 32]. Although *Intestinibacter* was more abundant in early-stage breast cancer patients, this genus has not been directly linked to breast cancer but is known to be associated with insulin resistance and type 2 diabetes [33], which are known breast cancer risk factors.

The presence of *Fusobacteria*, although not statistically significant in terms of prevalence, was notably enriched in the breast cancer group, especially in HER2-negative patients. Given its reported association with gastrointestinal cancers, this may imply a potential role in the development of typical HER2-negative breast cancer [28].

In terms of microbial diversity, the breast cancer cohort exhibited a significantly lower number of total bacterial species but higher uniformity. This shift was associated with a predicted reduction in microbial functional genes, enzymes, and pathways essential for maintaining host health and homeostasis, as inferred through PICRUSt2 analysis. Such dysbiosis may impair systemic equilibrium and facilitate breast cancer development.

Prior studies have also indicated associations between microbial diversity and breast cancer. A pilot study found that gut microbiota diversity differed between postmenopausal breast cancer patients and healthy individuals [29]. Additionally, a case–control study reported reduced microbial diversity and an increased Firmicutes-to-Bacteroidota ratio in HR-positive/HER2-negative breast cancer patients [30]. These findings are consistent with our results, suggesting that even subtle disruptions in gut microbiota diversity may contribute to disease onset.

When comparing subtypes, no consistent patterns were observed between healthy and cancer groups. However, HR-positive breast cancers, typically more differentiated, showed higher alpha diversity in species-weighted indices, indicating that microbial richness might influence tumor differentiation. Significant differences in beta diversity were noted based on menopausal status and lymph node metastasis, suggesting that gut microbiota may affect disease progression. Interestingly, the *Prevotellaceae NK3B31 group* was enriched exclusively in the HER2-type group, and though few studies have addressed this genus, it has been associated with glucose intolerance and obesity [34, 35], potentially linking metabolic factors with breast cancer subtypes.

This study has several limitations. One limitation is the relatively small sample size, which may have limited our ability to detect more subtle or subtype-specific microbiota differences. Based on prior data and assuming a statistical power of 80% and a significance level of α = 0.05, an estimated effect size (Cohen’s d) of approximately 0.35 would require around 130 participants per group to detect significant differences. However, due to constraints in sample collection, the current study included fewer participants, which limits the statistical power and generalizability of our findings. Another is the focus on a single ethnicity—Japanese women—whose gut microbiota composition differs from other populations [27], limiting the generalizability of our findings. Additionally, BMI afe known to significantly influence gut microbiota composition. However, BMI information was not clearly available for the PC cohort, and it is possible that the breast cancer group had a higher average BMI. This discrepancy may have introduced an important confounding factor affecting the observed microbial differences. Moreover, participants were randomly selected from each cohort rather than matched using methods such as propensity score matching, and as such, age was not strictly controlled between groups. Given that age can influence gut microbiota, this may have introduced additional confounding. Furthermore, due to the cross-sectional design, we cannot determine whether the observed gut microbiota differences are a cause or a consequence of breast cancer. Moreover, our functional predictions were based on 16S rDNA profiles analyzed via PICRUSt2, which does not directly measure genes or metabolites and thus cannot fully reflect the actual metabolic capabilities of the microbiota.

Although this represents a substantial limitation, the clear trends observed between healthy and cancer groups may still capture meaningful biological shifts associated with disease states.

In conclusion, our findings support the hypothesis that dysbiosis of the gut microbiota is associated with breast cancer. Targeting gut microbiota composition and function could serve as a novel avenue for breast cancer prevention and management.

## Data Availability

DNA sequences corresponding to the 16S rRNA gene in the NIBIOHN cohort have been deposited in DDBJ under accession numbers DRA010839 (Minamiuonuma, Niigata, Japan), DRA010837 and DRA010838 (Shinjuku, Tokyo, Japan), DRA010840 and DRA014928 (Osaka-shi, Osaka, Japan), DRA012134, DRA010841 and DRA015021 (Shunan, Yamaguchi, Japan) ((https://ddbj.nig.ac.jp) DNA sequences data of the breast cancer cohort have not been made public because of ethical considerations and the need to protect patient privacy and confidentiality.
